# Impact of emergency medical service with advanced life support training for adults with out-of-hospital cardiac arrest in the Republic of Korea: A retrospective multicenter study

**DOI:** 10.1371/journal.pone.0286047

**Published:** 2023-06-08

**Authors:** Jae Yun Ahn, Hyun Wook Ryoo, Haewon Jung, Young Sun Ro, Jeong Ho Park

**Affiliations:** 1 Department of Emergency Medicine, School of Medicine, Kyungpook National University, Daegu, Republic of Korea; 2 Laboratory of Emergency Medical Services, Seoul National University Hospital Biomedical Research Institute, Seoul, Republic of Korea; 3 Department of Emergency Medicine, Seoul National University College of Medicine and Hospital, Seoul, Republic of Korea; Yokohama City University, JAPAN

## Abstract

Prehospital advanced life support (ALS) has been offered in many countries for patients experiencing out-of-hospital cardiac arrest (OHCA); however, its effectiveness remains unclear. This study aimed to determine the impact of emergency medical service (EMS) with ALS training as a nationwide pilot project for adults with OHCA in the Republic of Korea. This retrospective multicenter observational study was conducted between July 2019 and December 2020 using the Korean Cardiac Arrest Research Consortium registry. The patients were categorized into an intervention group that received EMS with ALS training and a control group that did not receive EMS with ALS training. Conditional logistic regression analysis was performed using matched data to compare clinical outcomes between the two groups. Compared with the control group, the intervention group had a lower rate of supraglottic airway usage (60.5% vs. 75.6%) and a higher rate of undergoing endotracheal intubation (21.7% vs. 6.1%, *P* < 0.001). In addition, the intervention group was administered more intravenous epinephrine (59.8% vs. 14.2%, *P* < 0.001) and used mechanical chest compression devices more frequently in prehospital settings than the control group (59.0% vs. 23.8%, *P* < 0.001). Based on the results of multivariable conditional logistic regression analysis, survival to hospital discharge (odds ratio: 0.48, 95% confidence interval: 0.27–0.87) of the intervention group was significantly lower than that of the control group; however, good neurological outcome was not significantly different between the two groups. In this study, survival to hospital discharge was worse in patients with OHCA who received EMS with ALS training than in those who did not.

## Introduction

To date, out-of-hospital cardiac arrest (OHCA) has been recognized as a global public health issue associated with low survival and high disability rates [1]. Notably, its incidence and prognosis differ significantly among countries. In the United States, >350,000 adult cases of OHCA have been reported per year, with a survival to hospital discharge rate of 10.5% in 2018 [2]. In the Republic of Korea, approximately 30,000 cases of OHCA were reported in 2016, with a survival to hospital discharge rate of 7.6% [3]. Various efforts, such as public access defibrillation program in the community, increased rate of bystander cardiopulmonary resuscitation (CPR), and high-quality post-cardiac arrest care, have considerably improved OHCA outcomes [4–7]. The United States and other developed countries have implemented prehospital advanced life support (ALS) as an important strategy for the management of OHCA; however, its effectiveness remains debatable [8].

Prehospital use of epinephrine in patients with OHCA has been reported to improve the prehospital return of spontaneous circulation (ROSC) and increase 30-day survival [9,10]. However, its effects on long-term survival and neurological outcomes remain unknown [9,11,12]. Two large-scale randomized controlled trials have recently been conducted on the effectiveness of prehospital advanced airway management (AAM) in patients with OHCA. One study found no notable differences in outcomes between the use of a supraglottic airway (SGA) device and endotracheal intubation (ETI) [13], whereas another study reported that the use of a laryngeal tube (LT) resulted in a higher 72-h survival rate than when an ETI was performed [14]. However, in another subsequent study, the low quality of ETI was found to have possibly influenced the outcomes, considering the low first-pass success rate and high incidence of unsuccessful ETI [15]. In a prehospital ALS study in Ontario, although prehospital ALS improved ROSC, there was no improvement in the survival rate [16]. In addition, in subsequent OHCA studies [8,17], ALS did not demonstrate better outcomes than basic life support (BLS); however, these conclusions cannot be generalized to all settings.

Since July 2019, the Republic of Korea has been conducting a nationwide pilot project that provides dedicated EMS with ALS-centered resuscitation training. This study aimed to determine whether EMS with ALS training is associated with improved outcomes in adults with OHCA in the Republic of Korea.

## Materials and methods

### Study design and data source

This retrospective multicenter study included 64 participating hospitals in the Republic of Korea and was conducted between July 2019 and December 2020 using the Korean Cardiac Arrest Research Consortium (KoCARC) registry. To coordinate with studies in the area of OHCA resuscitation and increase the collaborative efforts for conducting such studies, KoCARC—a nationwide volunteer-based research network—was developed. The KoCARC registry includes data of patients with OHCA who were transferred by the EMS team to the emergency department (ED) of the participating hospitals with ongoing resuscitation efforts, with the medical etiology of OHCA being identified by emergency physicians in each ED [18]. Patients who had a terminal illness, received hospice care, were pregnant, had do-not-attempt resuscitation cards, or had presented for nonmedical causes (such as trauma, drowning, asphyxia, burns, hanging, or poisoning) were excluded from the study. A web-based electronic database registry was used to collect and store KoCARC data using a defined registry form. Each participating ED assigned a local research coordinator to oversee the review of records and ensure data accuracy. A quality management committee was established to review the completeness and consistency of the mandatory data variables and to provide regular feedback to the research coordinators and investigators regarding the results of the quality management processes.

### Study setting

The National Fire Agency is in charge of the Republic of Korea’s government-based public EMS system. As of 2018, it had 18 provincial fire headquarters, 219 fire stations, 1,055 safety centers, and 1,420 ambulances. Certified EMS providers included level 1 emergency medical technicians ([EMTs]; comparable to an advanced EMT in the USA), level 2 EMTs (similar to an EMT), and registered nurses. Before the project, a level 1 EMT could administer AAM and intravenous (IV) access to patients with OHCA under online medical oversight via a cellular phone call. However, such IV drug administration is not legally permitted in the Republic of Korea, according to the Enforcement Rule of the EMS Act. When the emergency medical dispatcher recognized the emergency call as a cardiac arrest, a multi-tiered, dual-dispatch system was used to dispatch two EMS teams to the scene, arriving via two ambulances or one ambulance with one fire engine, depending on the proximity and availability of resources.

### Study population

This study included adult patients (aged ≥18 years) with OHCA caused by medical etiologies. Patients with OHCA witnessed by EMS; those who were transferred from other hospitals; and those who had missing or incomplete information about the bystander’s CPR status, witness status, initial electrocardiogram (ECG) rhythm, and urbanization level of the cardiac arrest location were excluded from the study.

### Study intervention

In the Republic of Korea, one EMS unit per fire station was designated for 24-h availability during the project (hereafter referred to as advanced EMS) and comprised three EMS providers, including at least two level 1 EMTs or nurses. In addition to OHCA, advanced EMS was also dispatched to the scene at the request of the dispatch center in cases of suspected major trauma, anaphylaxis, ischemic chest pain and emergency delivery. When OHCA was detected, the emergency medical dispatcher initiated a dual-dispatch system, ideally including the advanced EMS if one was available.

Advanced EMS included the following interventions: first, level 1 EMTs and nurses among its members were required to complete the newly developed CPR training course for the project, which included a theory session on utilizing IV access, epinephrine administration, usage of a portable capnography device, AAM, and a simulation training session centered on team resuscitation at the field. Second, for the course of the trial, an advanced EMS provider was authorized to administer IV epinephrine to patients with OHCA while being closely monitored under medical director’s supervision via real-time video call. oversight. Third, upon arrival at the target hospital, feedback on prehospital resuscitation was obtained directly from the attending physicians.

### Matched data and variables

The study patients were categorized into an intervention group that received advanced EMS and a control group that did not receive advanced EMS depending on whether the dispatched EMS included an advanced EMS. Data were matched to ensure that the confounding factors were distributed equally throughout the control and intervention groups. The ratio of matching between the intervention and control groups was 1:1, with matching for sex, age (18–64, 65–74, and 75–120 years), urbanization level (metropolitan, urban, and rural), study period (second half of 2019, first half of 2020, and second half of 2020), and arrest location (public and nonpublic places).

The KoCARC registry provided the following data: demographics (sex, age, urbanization level, and arrest location); CPR-related characteristics, such as bystander CPR status, prehospital defibrillation, initial ECG rhythm, witness status, prehospital airway management, status of prehospital IV epinephrine use, and use of prehospital mechanical chest compression devices (MCDs); CPR-related time variables, such as response time interval (RTI), scene time interval (STI), and transport time interval (TTI); and clinical outcomes, such as prehospital ROSC, survival to hospital discharge, and neurological outcome. RTI was defined as the interval between an emergency call and an ambulance’s arrival at the scene. STI was defined as the time interval between the ambulance’s arrival at the scene and its departure from the hospital. TTI was defined as the period between the ambulance leaving the scene and arriving at the hospital.

### Outcome measurement

The primary outcome of this study was survival to hospital discharge. The secondary outcome was good neurological outcome at hospital discharge, defined as Cerebral Performance Category 1 or 2.

### Statistical analysis

All statistical analyses were performed using the R software version 4.0.5 (R Foundation for Statistical Computing, Vienna, Austria). Categorical variables were expressed as frequencies and percentages with Pearson’s chi-square test used for the analysis, while continuous variables were expressed as medians and interquartile ranges (IQR, 25^th^ and 75th percentiles) with the Mann–Whitney U-test because the data were positively skewed in the Shapiro–Wilk test.

The matched dataset was subjected to univariable and multivariable conditional logistic regression analyses to determine the impact of the intervention on the primary and secondary outcomes of OHCA as well as to calculate the adjusted odds ratio (aOR) and 95% confidence intervals (CIs) after adjusting for potential confounders for witness status, bystander CPR provision, prehospital defibrillation, initial rhythm, and RTI. To ascertain if the delayed arrival of advanced EMS had an impact on clinical outcomes, we performed supplementary analysis for clinical outcomes according to the RTI and response type of advanced EMS in intervention group. All statistical tests were two-tailed at a significance level of 0.05.

### Ethics statement

This study was reviewed and approved by the Kyungpook National University Hospital Institutional Review Board (2015-11-013-013), and the requirement for obtaining informed consent from the patients was waived. The study was registered at ClinicalTrials.gov (identifier: NCT03222999).

## Results

**Fig 1:**
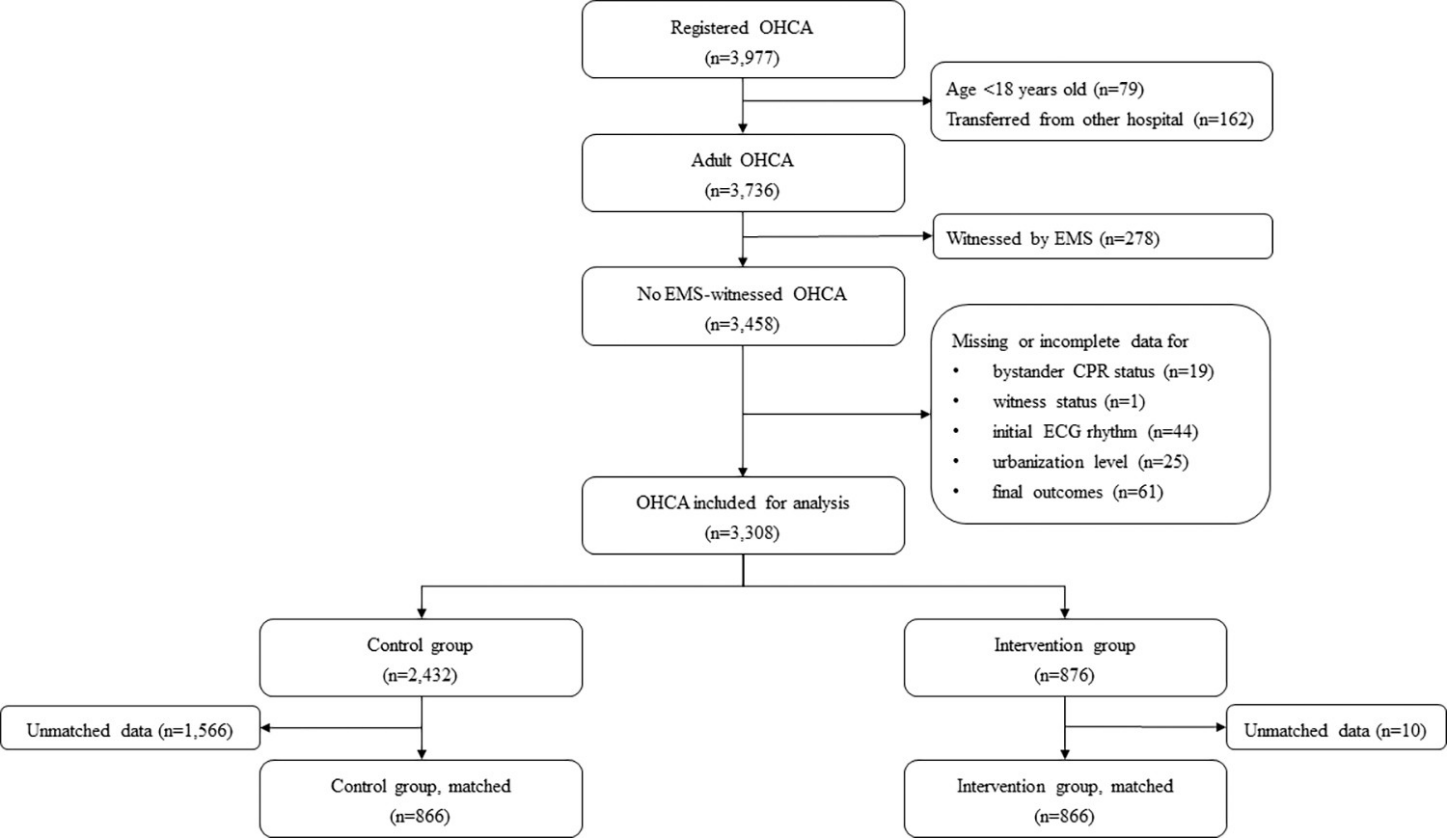
Flow diagram of the study. OHCA, out-of-hospital cardiac arrest; EMS, emergency medical services; CPR, cardiopulmonary resuscitation; ECG, electrocardiogram.

Overall, 3,977 patients were enrolled in the KoCARC registry during the study period. Of these, we excluded those aged <18 years (n = 79); transferred from other hospitals (n = 162); whose cardiac arrest was witnessed by EMS (n = 278); and who had missing or incomplete data on bystander CPR status, witness status, initial ECG rhythm, urbanization level, or final outcomes (n = 150). In total, 3,308 patients were included in the final analysis, with 876 (26.5%) patients in the intervention group. The intervention and control groups comprised 866 patients after 1:1 matching. Fig 1 shows the flow diagram of the study.

### Baseline characteristics and clinical outcomes of the study population

Table 1 provides an overview of the baseline characteristics and clinical outcomes of the study population both before and after data matching. Regardless of matching status, bystander CPR status, prehospital defibrillation, and initial ECG rhythm were not significantly different between the two groups. Before matching, the control group had a higher rate of witnessed cardiac arrest than the intervention group (54.6% vs. 48.7%, *P* = 0.004). However, after matching, there was no significant difference between the two groups (53.2% vs. 48.5%, *P* = 0.055). Based on the matched data, the intervention group had a higher rate of ETI (21.7% vs. 6.1%) and lower frequency of SGA usage than the control group (60.5% vs. 75.6%, *P* < 0.001). Moreover, the intervention group had a significantly higher rate of IV epinephrine administration (59.0% vs. 14.2%, *P* < 0.001) and MCD usage (59.8% vs. 23.8%, *P* < 0.001) than the control group. The median STI of the intervention group was longer than that of the control group (17 min vs. 15 min, *P* < 0.001).

**Table 1:** Demographics and baseline characteristics of the study population before and after matching.

	Before matching			After matching		
	Control (n = 2,432)	Intervention (n = 876)	*P* value	Control (n = 866)	Intervention (n = 866)	*P* value
Sex, male	1,626 (66.9)	584 (66.7)	0.951	581(67.1)	581(67.1)	1.000
Age			0.422			
18–64	883 (36.3)	310 (35.4)		310 (35.8)	310 (35.8)	
65–74	477 (19.6)	190 (21.7)		182 (21.0)	182 (21.0)	
75–120	1,072 (44.1)	376 (42.9)		374 (43.2)	374 (43.2)	
Urbanization level			<0.001			1.000
Metropolitan	1,777 (73.1)	739 (84.4)		732 (84.5)	732 (84.5)	
Urban	46 (1.9)	11 (1.3)		8 (0.9)	8 (0.9)	
Rural	609 (25.0)	126 (14.4)		126 (14.5)	126 (14.5)	
Time period			0.148			1.000
2019.07.01– 2019.12.31.	807 (33.2)	280 (32.0)		275 (31.8)	275 (31.8)	
2020.01.01– 2020.06.30.	823 (33.8)	328 (37.4)		326 (37.6)	326 (37.6)	
2020.07.01– 2020.12.31.	802 (33.0)	268 (30.6)		265 (30.6)	265 (30.6)	
Location of arrest			0.241			1.000
Nonpublic	1,996 (82.1)	735 (83.9)		727 (83.9)	727 (83.9)	
Public	436 (17.9)	141 (16.1)		139 (16.1)	139 (16.1)	
Bystander CPR, yes	1,511 (62.1)	537 (61.3)	0.695	547 (63.2)	532 (61.4)	0.488
Prehospital defibrillation	574 (23.9)	217 (24.8)	<0.001	160 (22.3)	214 (24.7)	0.257
Initial rhythm			0.911			0.735
Shockable	436 (17.9)	154 (17.6)		151 (17.4)	151 (17.4)	
PEA	517 (21.3)	182 (20.8)		192 (22.2)	179 (20.7)	
Asystole	1,479 (60.8)	540 (61.6)		523 (60.4)	536 (61.9)	
Witness status, yes	1,327 (54.6)	427 (48.7)	0.004	461 (53.2)	420 (48.5)	0.055
Airway management			<0.001			<0.001
ETI	120 (4.9)	189 (21.6)		53 (6.1)	188 (21.7)	
SGA	1,842 (75.7)	527 (60.2)		655 (75.6)	524 (60.5)	
BVM	470 (19.3)	160 (18.3)		158 (18.2)	154 (17.8)	
Prehospital IV Epinephrine, yes	372 (15.3)	523 (59.7)	<0.001	123 (14.2)	518 (59.8)	<0.001
Mechanical chest compression device			<0.001			<0.001
Yes	573 (23.6)	517 (59.0)		206 (23.8)	511 (59.0)	
No	1,720 (70.7)	351 (40.1)		611 (70.6)	347 (40.1)	
Unknown	139 (5.7)	8 (0.9)		49 (5.7)	8 (0.9)	
Dual-dispatch, yes	2,031 (83.5)	828 (94.5)	<0.001	740 (85.5)	820 (94.7)	<0.001
RTI	8.0 [6.0–11.0]	8.0 [6.0–10.0]	<0.001	8.0 [6.0–11.0]	8.0 [6.0–10.0]	<0.001
STI	14.0 [10.0–19.0]	17.0 [13.0–20.0]	<0.001	15.0 [11.0–18.0]	17.0 [13.0–20.0]	<0.001
TTI	10.0 [7.0–13.0]	8.0 [6.0–11.0]	<0.001	9.0 [7.0–13.0]	8.0 [6.0–11.0]	0.001

Values are presented as frequency (%) or median (interquartile range).

CPR: Cardiopulmonary resuscitation; PEA: Pulseless electrical activity; ETI: Endotracheal intubation; SGA: Supraglottic airway; BVM: Bag valve mask; IV: Intravenous; RTI: Response time interval; STI: Scene time interval; TTI: Transport time interval; ROSC: Return of spontaneous circulation.

### Clinical outcomes and conditional logistic regression analysis of the study population

Table 2 shows the results of the clinical outcomes and conditional logistic regression analysis of the intervention group compared with the control group. In terms of clinical outcomes in matched patients with OHCA, prehospital ROSC was not significantly different between the two groups (13.3% and 11.9% in the intervention and control groups, respectively; *P* = 0.426). However, the rates of survival to hospital discharge (12.2% vs. 8.3%, *P* = 0.009) and good neurological outcome (8.5% vs. 5.4%, *P* = 0.023) were significantly lower in the intervention group than in the control group, respectively. In the univariable analysis, the intervention group had lower rates of survival to hospital discharge (OR: 0.63; 95% CI: 0.45–0.87) and good neurological outcome (OR: 0.60, 95% CI: 0.40–0.90) than the control group. In the multivariable conditional logistic regression analysis, the rate of survival to hospital discharge was significantly lower in the intervention group than in the control group (aOR: 0.48, 95% CI: 0.27–0.87); however, good neurological outcome did not differ significantly between the groups (aOR: 0.56, 95% CI: 0.24–1.31).

**Table 2:** Clinical outcomes and conditional logistic regression analysis of outcomes in the intervention group compared with the control group.

	Before matching		After matching			
	n/N (%)	*P* value	n/N (%)	*P* value	OR (95% CI)	AOR^a^ (95% CI)
**Prehospital ROSC**		0.883		0.426		
Control	298/2,432 (12.2)		115/866 (13.3)		1.00	1.00
Intervention	105/876 (12.0)		103/866 (11.9)		0.87 (0.65–1.17)	1.11 (0.74–1.69)
**Survival to hospital discharge**		0.004		0.009		
Control	293/2,432 (12.0)		106/866 (12.2)		1.00	1.00
Intervention	74/876 (8.4)		72/866 (8.3)		0.63 (0.45–0.87)	0.48 (0.27–0.87)
**Good neurologic outcome**		0.008		0.023		
Control	202/2.432 (8.3)		72/866 (8.3)		1.00	1.00
Intervention	48/876 (5.5)		47/866 (5.4)		0.60 (0.40–0.90)	0.56 (0.24–1.31)

OR: Odds ratio; AOR: Adjusted odds ratio; CI: Confidence interval.

^a^Adjusted for witness status, bystander CPR provision, initial rhythm, prehospital defibrillation, and response time interval.

### Clinical outcomes according to RTI and response type of advanced EMS in the intervention group

The results of the clinical outcomes according to the RTI and response type of advanced EMS in the intervention group are presented in Table 3. In the adjusted model, survival to hospital discharge was significantly lower in the intervention group than in the control group, with later arrival of the advanced EMS (aOR: 0.36, 95% CI: 0.15–0.84). In contrast, there were no significant differences in other groups.

**Table 3:** Clinical outcomes of the intervention group for patients with out-of-hospital cardiac arrest in terms of the advanced emergency medical service unit’s response time interval and response type.

	Survival to hospital discharge			Good neurological outcome		
	Survival/death	OR (95% CI)	AOR^a^ (95% CI)	Good/poor	OR (95% CI)	AOR^a^ (95% CI)
**Response time**						
Control group	106/760	1.00	1.00	72/794	1.00	1.00
Intervention group						
RTI < 6 min	20/119	1.55 (0.72–3.30)	1.23 (0.40–3.84)	13/126	1.38 (0.55–3.42)	0.63 (0.13–3.17)
6 ≤ RTI < 12 min	38/544	0.52 (0.33–0.81)	0.63 (0.33–1.18)	26/556	0.54 (0.32–0.92)	0.88 (0.36–2.18)
RTI > 12 min	14/131	0.46 (0.22–0.94)	0.40 (0.14–1.12)	8/137	0.38 (0.15–0.96)	0.30 (0.06–1.48)
**Response type**						
Control group	106/760	1.00	1.00	72/794	1.00	1.00
Intervention group						
Advanced EMS first	51/539	0.75 (0.50–1.14)	0.88 (0.48–1.62)	35/555	0.74 (0.46–1.20)	0.77 (0.35–1.72)
Advanced EMS later	21/255	0.44 (0.25–0.79)	0.36 (0.15–0.84)	12/264	0.38 (0.17–0.81)	0.42 (0.11–1.59)

OR: Odds ratio; AOR: Adjusted odds ratio; CI: Confidence interval; RTI: Response time interval; EMS: Emergency medical services.

^a^Adjusted for witness status, bystander CPR provision, initial rhythm, and prehospital defibrillation.

## Discussion

The primary objective of this study was to determine the impact of EMS with ALS training in adult patients with OHCA in the Republic of Korea based on the data from the national OHCA registry. Based on an analysis adjusted for covariates, we found that the intervention group, which received support from EMS with ALS training, was associated with a significantly poorer survival to hospital discharge than the control group.

In this study, prehospital IV epinephrine administration by advanced EMS was permitted. Notably, in a recent meta-analysis of adults with OHCA, epinephrine treatment was associated with an improved survival to hospital discharge rate [11]. The most recent CPR guidelines emphasize the need for adult OHCA patients with a nonshockable rhythm to be administered epinephrine as soon as possible to increase their chances of survival [19]. In a previous study, delayed epinephrine administration was found to be negatively associated with survival to hospital discharge in patients with OHCA [20]. In the present study, patients in the intervention group had a higher rate of prehospital epinephrine administration. However, we could not identify the timing and dose of epinephrine administration because they were not recorded in the registry. Alternatively, in our supplementary analysis, we discovered that late arrival of advanced EMS was associated with a lower rate of survival to hospital discharge. The small number of advanced EMS units may not have achieved the early administration of epinephrine to patients in the intervention group during the study period owing to their late arrival, which may have negatively affected survival to hospital discharge.

Moreover, delayed epinephrine administration may be associated with longer STI, which is associated with poor outcomes for OHCA [20]. In the present study, STI was longer in the intervention group than in the control group. This may be attributed to the following reasons: First, emergency responders may have stayed longer and performed more complex resuscitation techniques. Second, a lack of advanced EMS units may have delayed the arrival of the EMS team at the scene. The lack of ALS providers in a tiered-response EMS system may result in a delay in the arrival of ALS providers [21]. Although the actual prehospital resuscitation time intervals after arrival at the scene in the delayed advanced EMS arrival group were comparable to those in the control group, this interval would have prolonged the STI and further delayed hospital arrival. Various time intervals for an optimal STI have been suggested in the literature. Nagao et al. [22] suggested that the prehospital resuscitation duration in OHCA should be extended by ≥33 min based on a Japanese population-based registry. However, another study suggested that the decision to transport the patient from the scene to the hospital should be made early during resuscitation as the survival rate of OHCA patients transported with continuous resuscitation was found to be decreased with an STI of >20 min [23]. In another study, the optimal STI was reported to be 8–15 min because 90% of survivors achieved a prehospital ROSC within the first 15 min of EMS resuscitation [24]. Depending on the initial ECG rhythm, whether the termination of resuscitation rule is applied at the scene, and the primary outcome, the optimal STI in OHCA varies from 4 to 50 min [25–27].

In addition, the availability of extracorporeal CPR (eCPR)-capable emergency centers should be considered in the local EMS protocols when determining prehospital resuscitation duration. Notably, rapid eCPR with a transfer time of <30 min enhanced survival to hospital discharge in OHCA patients with refractory ventricular fibrillation in a single-center randomized controlled trial [28]. According to the guidelines for adult eCPR, the time from cardiac arrest to establishment of adequate extracorporeal membrane oxygenation (ECMO) flow should be <60 min [29]. Therefore, if a patient is a potential ECMO candidate, the recommended total duration of resuscitation, including STI, should be considered in the local EMS protocols. Adults with OHCA should receive BLS only at the scene and be transported to the hospital as soon as possible if the duration of resuscitation is expected to exceed the currently recommended STI or if there is insufficient time to provide adequate ALS after the arrival of the advanced EMS team.

The use of ETI and MCDs was more common in the intervention group than in the control group. According to the latest guidelines for advanced airway placement in patients with OHCA, healthcare providers with sufficient experience in advanced airway placement can choose between ETI and SGA; alternatively, SGA is preferred [19]. Advantages of ETI include direct access to the lungs, assistance with ventilator and oxygenation regulation, and protection of the airway from aspiration to OHCA [30]. However, ETI generally requires more time to insert and is more complex and difficult to perform than SGA [13]. In a randomized clinical trial comparing LT and ETI, initial LT and ETI success rates were 90.3% and 51.6%, respectively, which revealed lower rates of ETI airway success [14]. Additionally, the success rate of ventilation with ETI was 79.0%, which was lower than that of ventilation with SGA (87.4%) [13]. ETI has been associated with potentially adverse effects, such as unrecognized esophageal intubation and excessive interruption of chest compressions [31,32]. Hence, to achieve the positive effect of ETI, proficiency in skills via adequate training is necessary. A previous study reported that ≥240 ETIs are needed to achieve a highly qualified ETI performance [33]. However, a study in Japan revealed that 95.7% of emergency life-saving technicians who received extensive training had an annual experience of performing ETI <2 times in a year, indicating that there were insufficient opportunities in the field [34]. The level of experience of EMTs with each method of AAM could not be determined in our study. In addition, proficiency parameters in AAM, such as success rate or number of attempts, were not recorded. The EMS team used their discretion in selecting the advanced airway type at the scene. It is important to provide accurate and clear criteria for equipment selection as knowledge and skills in AAM can affect the prognosis of OHCA.

Moreover, when deployed in the field, MCDs must be skillfully implemented, similar to AAM. The use of MCDs in OHCA has been associated with a reduced rate of survival to discharge in a meta-analysis of randomized controlled trials on MCDs [35]. Major factors that adversely affect the use of an MCD include the interruption and delay in chest compressions for deployment of the device and the delay in first defibrillation for OHCA with a shockable rhythm. Nevertheless, a previous study demonstrated that the use of MCDs reduced the no-flow fraction time from 35% to 16% during resuscitation [36]. This result suggests that the benefit of reducing interruptions in chest compressions during resuscitation until arrival at the hospital can be achieved if EMS providers are well trained to avoid interruptions in CPR during the first MCD deployment. In the present study, the use of MCDs differed between the two groups; however, MCD skills training were not included in some parts of the CPR training course. Therefore, team resuscitation training should be strengthened by including workshops for EMS providers on the usage of MCDs at the scene.

The results of this study indicate that some prehospital intervention components need to be improved and revised. First, STIs should be included in the field protocol and monitored continuously to avoid unnecessarily increasing the time to arrival in ED. Second, training for field ALS skills should be increased and priority to each skill performed in the field should be specified. Furthermore, the lack of an advanced EMS unit prevented several patients in the intervention group from receiving early ALS treatment. Thus, an appropriate expansion and organization of advanced EMS should be considered to provide further appropriate early prehospital ALS.

This study has some limitations. First, during the study period, the coronavirus disease 2019 (COVID-19) pandemic began in early 2020 in the Republic of Korea. Although the study period of the three intervals was matched equally between the two groups, the pandemic may have affected the clinical outcomes of patients with OHCA depending on the presence of suspected COVID-19 symptoms or the surge of COVID-19 cases in a specific region. Second, the performance and quality of ALS procedures affecting the outcomes of OHCA, such as the success rate, number of ETI attempts by EMS providers, and CPR performance, were not measured because they were not included in the registry.

## Conclusions

In this study, survival to hospital discharge was significantly worse in adult patients with OHCA in the Republic of Korea who received EMS with ALS training than those who did not.
